# Low Systemic Levels of Chemokine C-C Motif Ligand 3 (CCL3) are Associated with a High Risk of Venous Thromboembolism in Patients with Glioma

**DOI:** 10.3390/cancers11122020

**Published:** 2019-12-14

**Authors:** Pegah Mir Seyed Nazari, Christine Marosi, Florian Moik, Julia Riedl, Öykü Özer, Anna Sophie Berghoff, Matthias Preusser, Johannes A. Hainfellner, Ingrid Pabinger, Gerhard J. Zlabinger, Cihan Ay

**Affiliations:** 1 Division of Hematology and Hemostaseology, Department of Medicine I and Comprehensive Cancer Center Vienna, Medical University of Vienna, 1090 Vienna, Austria; pegah.mirseyednazari@meduniwien.ac.at (P.M.S.N.); Florian.moik@meduniwien.ac.at (F.M.); julia.riedl@meduniwien.ac.at (J.R.); oyku.oezer@gmail.com (Ö.Ö.); ingrid.pabinger@meduniwien.ac.at (I.P.); 2 Division of Oncology, Department of Medicine I and Comprehensive Cancer Center Vienna, Medical University of Vienna, 1090 Vienna, Austria; christine.marosi@meduniwien.ac.at (C.M.); anna.berghoff@meduniwien.ac.at (A.S.B.); matthias.preusser@meduniwien.ac.at (M.P.); 3 Institute of Neurology and Comprehensive Cancer Center Vienna, Medical University of Vienna, 1090 Vienna, Austria; johannes.hainfellner@meduniwien.ac.at; 4 Institute of Immunology, Center of Pathophysiology, Infectiology and Immunology, Medical University of Vienna, 1090 Vienna, Austria; gerhard.zlabinger@meduniwien.ac.at; 5 I.M. Sechenov First Moscow State Medical University (Sechenov University), 119146 Moscow, Russia

**Keywords:** venous thromboembolism, glioma, inflammation, cytokines, CCL3

## Abstract

A tight interplay between inflammation and hemostasis has been described as a potential driver for developing venous thromboembolism (VTE). Here, we investigated the association of systemic cytokine levels and risk of VTE in patients with glioma. This analysis was conducted within the prospective, observational Vienna Cancer and Thrombosis Study. Patients with glioma were included at time of diagnosis or progression and were observed for a maximum of two years. Primary endpoint was objectively confirmed VTE. At study entry, a single blood draw was performed. A panel of nine cytokines was measured in serum samples with the xMAP technology developed by Luminex. Results: Overall, 76 glioma patients were included in this analysis, and 10 (13.2%) of them developed VTE during the follow-up. Chemokine C-C motif ligand 3 (CCL3) levels were inversely associated with risk of VTE (hazard ratio [HR] per double increase, 95% confidence interval [CI]: 0.385, 95% CI: 0.161–0.925, *p =* 0.033), while there was no association between the risk of VTE and serum levels of interleukin (IL)-1β, IL-4, IL-6, IL-8, IL-10, IL-11, tumor necrosis factor (TNF)-α and vascular endothelial growth factor (VEGF), respectively. In conclusion, low serum levels of CCL3 were associated with an increased risk of VTE. CCL3 might serve as a potential biomarker to predict VTE risk in patients with glioma.

## 1. Introduction

Venous thromboembolism (VTE), defined as deep vein thrombosis (DVT) and pulmonary embolism (PE), can be a life-threatening condition that frequently occurs in patients with cancer [1,2,3]. Interestingly, a number of inflammatory and hemostatic biomarkers have been found to be associated with risk of VTE in patients with glioma [4,5]. A mechanistic link between inflammation and VTE has been discussed [6,7]. Inflammatory cells as well as cytokines are able to activate the hemostatic system, and thereby promoting a hypercoagulable state [8]. Upon inflammatory activation, cytokines can be released by different cell types and vice versa act also on different cell types [9]. Cytokines are able to activate the coagulation cascade directly or indirectly through activation of neutrophils or monocytes, which have been described to play a role in thrombus formation [8,10,11,12]. Moreover, tumor cells themselves are able to interact with the immune system via several pathways, which can lead to thrombus formation [13,14]. Additionally, tumor cells are able to release cytokines, and elevated cytokine levels have been observed in patients with various cancer entities [15,16,17].

Primary brain tumors belong to those tumor entities with a particularly high VTE risk, and 10% to 20% of the patients develop VTE during the course of the disease [18]. Previously, several risk factors have been described [19,20,21,22,23]. However, the impact of anti- and pro-inflammatory cytokines on thromboembolic events in patients with glioma has not been evaluated in detail. Therefore, in this exploratory study, we aimed to investigate the association of various cytokine levels with the risk of future VTE in patients with glioma included in a prospective observational cohort study.

## 2. Results

### 2.1. Baseline Characteristic of the Study Population

In this study, 76 glioma patients (median age: 54 years (interquartile range (IQR): 46–67 years), 46.1% female), for whom serum samples were available, were included. In total, 57/76 (75%) were diagnosed with glioblastoma (World Health Organization [WHO] grade IV), 14/76 (18.4%) with anaplastic glioma (WHO grade III) and 5/76 (6.6%) with diffuse glioma (WHO grade II). Overall, 14/76 (18.4%) glioma had an isocitrate dehydrogenase 1 (IDH1) mutation. The detailed characteristics of the study population are summarized in Table 1.

### 2.2. VTE Events during Follow-Up

Patients were followed for a median of 358 days (IQR: 185–600 days) for occurrence of VTE. During the observation time, 10/76 (13.2%) patients with glioma developed VTE. The cumulative 6-, 12- and 24-month probability of VTE was 10.2%, 15.6%, and 15.6%, respectively.

Estimates in competing risk analysis were slightly lower, with a cumulative incidence of 9.3%, 13.4%, and 13.4% after 6, 12, and 24 months, respectively.

### 2.3. Serum Cytokine Levels and Risk of VTE

The distribution of cytokine levels (pg/mL) in the study population is listed in Table 1. In univariable cox regression analysis, chemokine C-C motif ligand 3 (CCL3) levels were significantly associated with risk of developing VTE (hazard ratio [HR] per double increase, 95% confidence interval [CI]: 0.385, 95% CI: 0.161–0.925, *p* = 0.033), while no significant association with VTE was found for serum levels of interleukin (IL)-1β (HR per double increase: 1.046, 95% CI: 0.732–1.495, *p* = 0.804), IL-4 (0.907, 95% CI: 0.634–1.298, *p* = 0.593), IL-6 (0.894, 95% CI: 0.602–1.3628, *p* = 0.597), IL-8 (0.918, 95% CI: 0.585–1.442, *p* = 0.711), IL-10 (0.822, 95% CI: 0.505–1.340, *p* = 0.433), IL-11 (0.621, 95% CI: 0.284–1.357, *p* = 0.232), tumor necrosis factor (TNF)-α (1.097, 95% CI: 0.697–1.727, *p* = 0.689), and soluble vascular endothelial growth factor (VEGF) (0.995, 95% CI: 0.640–1.548, *p* = 0.983), respectively (Table 2).

In multivariable analysis, adjusting for potential confounders such as sex, age and WHO grade IV, the negative association of CCL3 with the risk of VTE remained statistically significant (CCL3: adjusted HR per double increase: 0.329, 95% CI: 0.128–0.843, *p =* 0.021) (Table 3). In a second multivariable model, we additionally adjusted for biomarkers previously identified to predict risk of cancer-associated VTE (e.g., platelet count, sP-selectin and D-dimer), and the association of lower CCL3 levels with higher risk of VTE remained statistically significant (CCL3: adjusted HR per double increase: 0.203, 95% CI: 0.059–0.694, *p =* 0.011). Furthermore, we adjusted for positive podoplanin expression on tumor samples (CCL3: adjusted HR per double increase: 0.384, 95% CI: 0.146–0.830, *p =* 0.017) and IDH1 mutation status (CCL3: adjusted HR per double increase: 0.412, 95% CI: 0.174–0.975, *p =* 0.044), and the inverse association of serum CCL3 levels with risk of glioma-associated VTE prevailed.

### 2.4. Low Levels of Systemic CCL3 Predict the Risk of VTE

**Figure 1:**
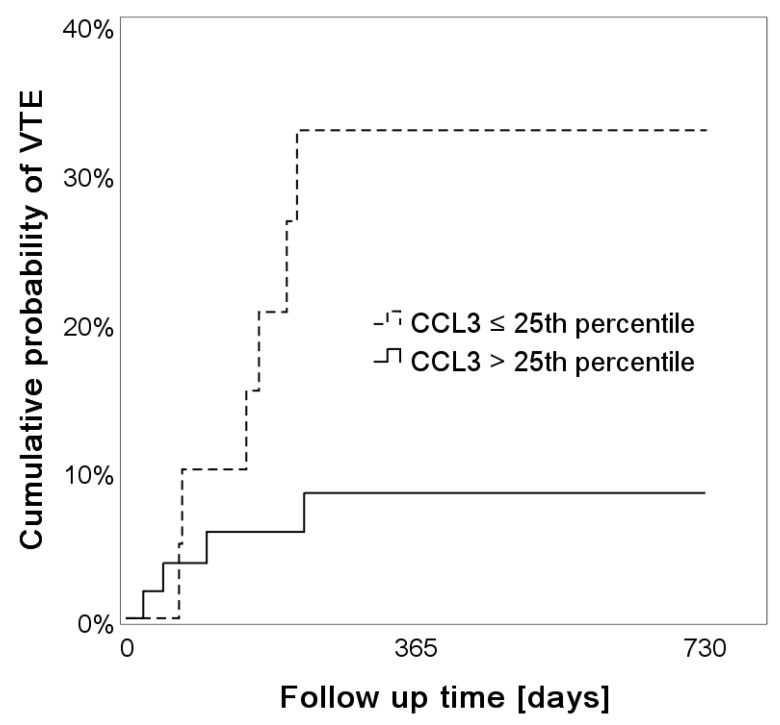
Chemokine C-C-motif ligand 3 (CCL3) and risk of venous thromboembolism (VTE) in patients with glioma: low serum levels of CCL3 were significantly associated with a higher risk of VTE (log-rank test, *p =* 0.019).

In order to identify distinct VTE risk profiles, we defined two groups of patients, based on low and high systemic CCL3 levels. The cutoff was chosen at the 25th percentile. In reverse Kaplan-Meier analysis, the 6-, 12- and 24- month cumulative probability of VTE was 20.6%, 32.8% and 32.8% in patients with low CCL3 serum levels compared to 5.8%, 8.4% and 8.4% in those with higher CCL3 serum levels (log-rank test, *p* = 0.019), (Figure 1). These results were similar in competing risk analysis: the 24-month cumulative incidence was 30% (95%CI: 12.3–50.1) in patients with low CCL3 levels and 7.4% (95%CI: 2.4–16.3) in patients with high CCL3 levels.

During the follow-up time of two years, patients with low serum levels of CCL3 were associated with an increased risk of VTE compared to those with high systemic CCL3 levels (HR: 4.069, 95% CI: 1.147–14.438, *p* = 0.030) and this association prevailed upon adjustment for sex, age and WHO grade IV (adjusted HR: 4.552, 95% CI: 1.242–16.683, *p* = 0.022). These results were also confirmed in competing risk analysis: the subdistribution HR (sHR) for VTE was 4.46 (95%CI: 1.27–15.71, *p =* 0.020) and prevailed upon adjustment for sex, age and WHO grade IV (adjusted sHR: 4.891, 95% CI: 1.429–16.739, *p =* 0.011).

### 2.5. Association of Systemic Cytokine Levels and Tumor Grade

**Figure 2:**
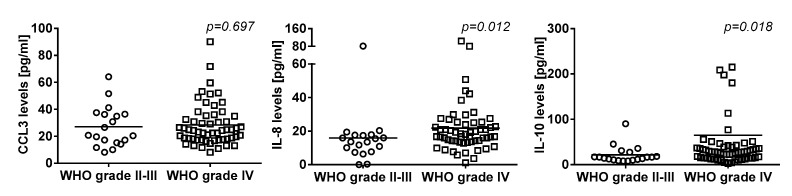
Serum cytokine levels and WHO tumor grade (II-III vs. IV) in patients with glioma. (CCL3 = chemokine C-C motif ligand 3, IL-8 = interleukin-8, IL-10 = interleukin-10).

Serum cytokine levels and their association with tumor grade in our glioma cohort are summarized in Table 4. In detail, there was no difference of systemic levels of CCL3 according to the tumor grade (WHO grade IV vs. grade II-III, median (IQR): 24.3 (17.8–35.1) vs. 20.7 (15.2–36.4) pg/mL, *p* = 0.232), while systemic levels of IL-8 (18.1 (18.1–25.2) vs. 13.6 (7.7–17.6) pg/mL, *p =* 0.012) and IL-10 (28.4 (14.5–40.6) vs. 16.6 (10.7–27.3) pg/mL, *p* = 0.018) were higher in patients with WHO grade IV tumors compared to WHO grade II-III tumors (Figure 2). Also, systemic levels of IL-1β (1.9 (0.6–3.4) vs. 1.9 (1.1–4.8) pg/mL, *p =* 0.211), IL-4 (11.5 (9.5–26.6) vs. 11.5 (11.5–37.3) pg/mL, *p =* 0.536), IL-6 (3.8 (1.8–9.8) vs. 3.8 (1.4–8.4) pg/mL, *p =* 0.719), IL-11 (0 (0–5.9) vs. 0 (0–37.2) pg/mL, *p =* 0.249), TNF-α (11.7 (6.7–20.9) vs. 11.4 (7.8–17.1) pg/mL, *p =* 0.540) and VEGF (137.0 (81.7–241.1) vs. 160.8 (81.7–376) pg/mL, *p =* 0.819), respectively, were not significantly different between tumor grades.

## 3. Discussion

In the current study, we analyzed a panel of cytokines and found that lower serum levels of the chemokine CCL3 were associated with an elevated risk of VTE in patients with glioma. In contrast, systemic levels of IL-1β, IL-4, IL-6, IL-8, IL-10, IL-11, TNF-α, and VEGF, respectively, were not associated with the occurrence of VTE. Furthermore, we observed that systemic levels of IL-8 and IL-10 differed according to the tumor grade, with higher levels in patients with glioblastoma (WHO grade IV) compared to lower grade glioma (WHO grade II–III).

CCL3 is a member of the CC chemokine family and functions as a chemoattractant for various immune cells during acute inflammation [24]. Furthermore, CCL3 is inducible in most mature hematopoietic cells upon distinct regulators, such as LPS, TNF-α or IL-1β, whereas it is inhibited by IL-4 and IL-10 [24,25]. Previously, Collins et al. reported that CCL3 levels were inversely linked to overall coagulability and clotting strength (both measured via thromboelastography) in patients with primary Sjörgen’s syndrome [26]. This study supports an inverse association between CCL3 levels and hypercoagulability, which is in line with our finding of an association of lower systemic CCL3 levels with higher risk of VTE occurrence in patients with glioma. Interestingly, Kawao et al. demonstrated in a mouse model that mRNA levels of CCL3 were decreased in damaged femurs of mice deficient for plasminogen or urokinase-type plasminogen activator (uPA) during bone repair indicating that CCL3 might be induced by the tissue fibrinolytic system as well [27]. So far, we can only speculate how low serum levels of CCL3 might be mechanistically linked to a higher risk of VTE in patients with glioma. Whether an association of CCL3 with impaired fibrinolysis or vice versa lead to an increased risk of VTE needs to be further explored in a dedicated study.

The inverse association of CCL3 with VTE in our study was independent of previously described VTE risk factors in brain tumors [19,20,21,22,23]. For example, a higher glioma grade as well as distinct local tumor characteristics, such as the upregulation of podoplanin expression on tumor cells, are associated with a higher VTE risk [19]. Furthermore, an IDH1 wildtype status is linked to a higher risk of VTE, while patients with an IDH1 mutation have a very low VTE risk [20,21]. In addition, laboratory parameters, such as a low platelet count, a high leukocyte count and higher soluble P-selectin and D-Dimer levels, have been reported to be associated with a higher VTE risk in a larger cohort of patients with glioma [22]. Of note, in the present glioma patient cohort, D-dimer levels were not associated with VTE which is likely due to the low number of patients.

In the brain, CCL3 can be also upregulated by astrocytes and microglia [25]. In glioma, the presence of CCL3 in the tumor tissue was found to correlate with a higher tumor grade [28]. Of note, systemic levels of CCL3 levels did not correlate with the tumor grade in our glioma patient cohort. Whether local CCL3 concentrations in the tumor microenvironment might impact the occurrence of thromboembolic events has not been investigated.

Cytokines have the ability to interact with the hemostatic system via several pathways. The upregulation of tissue factor (TF), the main activator of coagulation in vivo, can be induced by pro-inflammatory cytokines (e.g., TNF-α, Il-1β, IL-6, IL-8) and inhibited by anti-inflammatory cytokines (e.g., IL-4, IL-10) [6,7,10,12,29,30,31,32,33]. Furthermore, cytokines (e.g., IL-8) can trigger the release of so-called neutrophil extracellular traps (NETs) by neutrophils, which can promote the formation of a thrombus [11,12,34,35,36]. Nonetheless, in contrast to CCL3, we found no association of VTE with other cytokines of the investigated panel (e.g., IL-1β, IL-4, IL-6, IL-8, IL-10, IL-11, TNF-α, VEGF) in our glioma cohort.

In patients without cancer, elevated levels of distinct pro-inflammatory cytokines (e.g., IL-6, IL-8) have been associated with the risk of VTE [6,37,38,39,40,41,42]. Previous studies also reported an association of several cytokines (e.g., IL-6) with cancer-associated VTE [43]. In pancreatic cancer, systemic levels of IL-1β and IL-6 showed a trend towards the development of future thrombosis [44]. In a large cohort of cancer patients, systemic levels of VEGF, an angiogenic factor that stimulates the development of highly thrombogenic vessels, were linked to a higher risk of VTE [45,46,47]. In patients with abdominal tumors, lower levels of the anti-inflammatory cytokine IL-10 were found in patients with DVT compared to those without DVT [48].

In the pathogenesis of cancer, cytokines are mechanistically involved in the promotion of tumor development, growth and invasion [49]. Interestingly, Ahlbrecht et al. demonstrated that a higher tumor grade increased the risk of cancer-associated VTE, including patients with glioma [19,50]. In the current study, we found that IL-8 and IL-10 levels were elevated in patients with a higher grade of glioma (WHO grade IV vs. grade II–III). However, we found no direct correlation of IL-8 or IL-10 with risk of developing VTE in our cohort of patients with glioma. However, as the samples size of the cohort was relatively small, our study might lack statistical power to detect a potential association of these cytokines with risk of VTE in patients with glioma.

Some other limitations of our study need to be addressed. Due to the exploratory and hypothesis-generating nature of this study, we did not correct for multiple testing. Furthermore, the cut-off for elevated systemic CCL3 at the quartiles was chosen arbitrarily. However, as no data are available on serum CCL3 reference ranges or clinically validated cutoffs, we used the cutoff at the 25th percentile, which has been commonly used in previous studies [51]. Due to our small patient cohort, we propose future studies with larger patient numbers in order to confirm our exploratory study. An open question remains, whether CCL3 might have direct inhibitory functions on coagulation or platelet activation or whether it may protect indirectly from thromboembolic events. Experimental studies are required to provide mechanistic explanations for our observation and to reveal potential direct or indirect effects of CCL3 on hemostasis and thrombosis.

## 4. Materials and Methods

### 4.1. Vienna Cancer and Thrombosis Study (CATS)

This study was conducted within the framework of the Vienna Cancer and Thrombosis Study (CATS), a prospective observational single-center cohort study, which has been initiated at the Medical University of Vienna (Vienna, Austria) with the aim to identify risk factors for VTE in patients with cancer. The study design has been described previously in detail [19,52]. Briefly, patients included in this analysis were recruited at time of glioma diagnosis or at time of progression after tumor resection, and were then observed for a maximum time period of 2 years. The primary endpoint of the study was objectively confirmed VTE. Exclusion criteria were clinically overt infection, thromboembolic events within the last 3 months, and continuous anticoagulation. Patients who underwent surgery or radiotherapy within the past 2 weeks before recruitment, and/or chemotherapy within the past 3 months were ineligible. Data on IDH1 mutation status and podoplanin expression in the tumor tissue as well as sP-selectin and D-dimer levels in the blood were available from our previous investigations [19,21,22,52,53].

The CATS study has been approved by the institutional ethics committee and has been conducted according to the declaration of Helsinki (EC number: 126/2003, ethik-kom@meduniwien.ac.at).

### 4.2. Blood Sampling

At study inclusion and prior to chemotherapy, venous blood samples were collected via sterile venipuncture in vacuum tubes (Vacuette, Greiner BioOne, Kremsmünster, Austria). Serum samples were obtained by centrifugation at 3600× *g* for 10 min and stored at −80 °C until measurements were conducted in series.

### 4.3. Serum Cytokine Levels

Serum concentrations of several cytokines were determined using commercially available Multiplex Bead-Based Kits (#HCYTOMAG-60K, #HCYP3MAG-63K, Merck, Millipore, Billerica, MA, USA) according to the manufacturer’s instructions. These multiplex immunoassays are based on the Luminex xMAP technology, which provides a fluorescent bead-based system that allows the simultaneous analysis of multiple cytokines and chemokines. Following cytokines and chemokines were measured in serum samples: CCL3, IL-1β, IL-4, IL-6, IL-8, IL-10, IL-11, TNF-α and VEGF. In brief, the capture antibody-coated beads were incubated with 25 μL of serum in a 96-well plate at 4 °C overnight with shaking. After incubation, the plate was washed three times. Then, detection antibodies were added and incubated for 1h at room temperature on a shaker. The reaction mixture was detected by Streptavidin–Phycoerythrin and incubated for 30 min at room temperature on a shaker. Again, the plate was washed three times and the beads were resuspended in sheath fluid for 5 min on a plate shaker. Plates were read on a Luminex 200 system (Luminex, Austin, TX, USA) and analyzed with the xPONENT 3.1. software (Luminex, Austin, TX, USA) using a four-parameter model to calculate final concentrations and values (expressed in pg/mL). All patient samples were measured undiluted and were run blinded to clinical data and information on VTE.

### 4.4. Statistical Analysis

Statistical analyses were performed using IBM SPSS Statistics (Version 2014, IBM Corp., Armonk, NY, USA) and Stata 14.0 (Stata Corp., Houston, TX, USA). Continuous variables were summarized as median [IQR], and categorical variables as absolute frequencies (%). The non-parametric Mann-Whitney U test was applied to compare differences between continuous variables that were not normally distributed. Median follow-up was estimated with the reverse Kaplan-Meier method. The probability of VTE was calculated with 1-Kaplan-Meier estimators. VTE incidences between groups were compared with log-rank tests. Hazards of VTE were modelled with uni- and multivariable cox regression models. We transformed the original cytokine measurements in order to provide risk estimates for a doubling of levels on any point of the individual scale (formula used for variable transformation: x_1_ = log(x)/log(2) [log = log with base-e]), which is a common strategy to provide comparable effect estimates among different markers with different measurement scales. To account for the significant risk of death in the cohort, cumulative incidences of VTE were additionally studied within a competing risks framework, treating death from any cause except fatal VTE as the competing event of interest [54]. In detail, the cumulative incidence of our thromboembolic endpoints were calculated with the competing risk estimator and associated standard errors as reported by Marubini & Valsecchi [55]. Uni- and multivariable modelling of time-to-event were performed with a proportional subhazards regression model according to Fine & Gray [56]. A *p*-value of <0.05 was defined as the cut-off for statistical significance. Due to the exploratory and hypothesis generating approach of the study, no adjustment for multiple testing was applied [57].

## 5. Conclusions

In this exploratory study, we demonstrated an inverse association between systemic CCL3 levels and risk of VTE in patients with glioma, and this observation was independent of important VTE predictors, such as tumor grade, and previously reported VTE-related systemic and local biomarkers in glioma, such as IDH1 mutation status and podoplanin expression on tumor cells. However, due to the small patient cohort, we propose to conduct larger cohort studies for validation of our results. In contrast, there was no association of systemic cytokine levels of IL-1β, IL-4, IL-6, IL-8, IL-10, IL-11, TNF-α and VEGF with risk of VTE in our patient cohort. Taken together, CCL3 might be an interesting and a novel inflammatory biomarker that might be useful in predicting the risk of VTE in patients with glioma. Further studies are needed to expand on our observation and confirm the inverse association of CCL3 and risk of VTE in patients with glioma.

## Figures and Tables

**Table 1:** Patient characteristics and systemic cytokine levels in the study population.

	Median (Interquartile Range) or Count (%)
Age	54 (46–67)
Female sex	35/76 (46.1%)
Newly-diagnosed glioma	63/76 (82.9%)
**IDH1 Status**	
IDH1 mutation	14/76 (18.4%)
IDH1 wildtype	62/76 (81.6%)
**Tumor Grade**	
WHO grade IV	57/76 (75%)
WHO grade III	14/76 (18.4%)
WHO grade II	5/76 (6.6%)
**Histology**	
Glioblastoma	56/76 (73.7%)
Gliosarcoma	1/76 (1.3%)
Anaplastic astrocytoma	12/76 (15.8%)
Anaplastic oligodendroglioma	2/76 (2.6%)
Diffuse astrocytoma	4/76 (5.3%)
Diffuse oligodendroglioma	1/76 (1.3%)
**Serum Cytokine Levels**	
IL-1β [pg/mL]	1.9 (0.8–3.7)
IL-4 [pg/mL]	11.5 (11.5–26.6)
IL-6 [pg/mL]	3.8 (1.6–8.9)
IL-8 [pg/mL]	16.7 (12.9–22.3)
IL-10 [pg/mL]	25.6 (13.5–36.6)
IL-11 [pg/mL]	0.0 (0.0–8.2)
CCL3 [pg/mL]	24.3 (17.2–35.8)
TNF-α [pg/mL]	11.7 (7.1–19.2)
VEGF [pg/mL]	143.1 (81.7–243.7)

**Table 2:** Univariable cox regression analyses of systemic cytokine levels and venous thromboembolism in patients with glioma.

Serum Cytokine Levels	HR *	95% CI		*p*-Value
CCL3 [pg/mL]	0.385	0.160	0.925	0.033
IL-1β [pg/mL]	1.046	0.732	1.495	0.804
IL-4 [pg/mL]	0.907	0.634	1.298	0.593
IL-6 [pg/mL]	0.894	0.602	1.328	0.597
IL-8 [pg/mL]	0.918	0.585	1.442	0.711
IL-10 [pg/mL]	0.822	0.505	1.340	0.433
IL-11 [pg/mL]	0.621	0.284	1.357	0.232
TNF-α [pg/mL]	1.097	0.697	1.727	0.689
VEGF [pg/mL]	0.995	0.640	1.548	0.983

* per double increase.

**Table 3:** Systemic chemokine C-C-motif ligand 3 (CCL3) levels and risk of glioma-associated VTE in multivariable cox regression analyses adjusted for potential confounders and previously established thrombotic risk factors.

**Model #1**	**Adjusted HR**	**95% CI**		***p*-Value**
CCL3 (pg/mL) ^1^	0.329	0.128	0.843	0.021
Sex	1.807	0.475	6.867	0.475
Age	1.028	0.971	1.088	0.341
Tumor grade ^2^	1.197	0.215	6.684	0.837
**Model #2**	**Adjusted HR**	**95% CI**		***p*-Value**
CCL3 (pg/mL) ^1^	0.203	0.059	0.694	0.011
D-dimer	0.811	0.632	1.042	0.102
sP-selectin	1.068	1.017	1.122	0.009
Platelets (G/L)	0.986	0.974	0.997	0.013
**Model #3**	**Adjusted HR**	**95% CI**		***p*-Value**
CCL3 (pg/mL) ^1^	0.348	0.146	0.830	0.017
Podoplanin ^3^	2.021	1.060	3.855	0.033
**Model #4**	**Adjusted HR**	**95% CI**		***p*-Value**
CCL3 (pg/mL) ^1^	0.412	0.174	0.975	0.044
IDH1 mutation ^4^	0.450	0.056	3.636	0.454

^1^ per double increase. ^2^ WHO grade IV vs. II-III. ^3^ Podoplanin expression (none, low, medium, high). ^4^ IDH1 mutation vs. IDH1 wildtype.

**Table 4:** Systemic cytokine levels and tumor grade (WHO grade II–III vs. IV) in patients with glioma.

Serum Cytokine Levels n = 76	WHO Grade II–III n = 19	WHO Grade IV n = 57	*p*-Value
CCL3 (pg/mL)	20.7 (15.2–36.4)	24.3 (17.8–35.1)	0.697
IL-1β (pg/mL)	1.9 (1.1–4.8)	1.9 (0.6–3.4)	0.211
IL-4 (pg/mL)	11.5 (11.5–37.3)	11.5 (9.5–26.6)	0.536
IL-6 (pg/mL)	3.8 (1.4–8.4)	3.8 (1.8–9.8)	0.719
IL-8 (pg/mL)	13.6 (7.7–17.6)	18.1 (18.1–25.2)	0.012
IL-10 (pg/mL)	16.6 (10.7–27.3)	28.4 (14.5–40.6)	0.018
IL-11 (pg/mL)	0 (0–37.2)	0 (0–5.9)	0.249
TNF-α (pg/mL)	11.4 (7.8–17.1)	11.7 (6.7–20.9)	0.540
VEGF (pg/mL)	160.8 (81.7–376)	137.0 (81.7–241.1)	0.819
